# Genus *Boswellia* as a new candidate for neurodegenerative disorders

**DOI:** 10.22038/IJBMS.2020.35288.8419

**Published:** 2020-03

**Authors:** Arezoo Rajabian, HamidReza Sadeghnia, Sahar Fanoudi, Azar Hosseini

**Affiliations:** 1 Pharmacological Research Center of Medicinal Plants, Mashhad University of Medical Sciences, Mashhad, Iran; 2 Department of Pharmacology, Faculty of Medicine, Mashhad University of Medical Sciences, Mashhad, Iran; 3 Division of Neurocognitive Sciences, Psychiatry and Behavioral Sciences Research Center, Mashhad University of Medical Sciences, Mashhad

**Keywords:** Alzheimer’s diseases, Boswellia, Cognitive, Neurodegenerative, diseases Neuroprotection

## Abstract

Neurodegenerative diseases, characterized by progressive loss of neurons, share common mechanisms such as apoptotic cell death, mitochondrial dysfunction, inflammation, and oxidative stress. Genus *Boswellia* is a genus in the Burseraceae family. It comprises several species traditionally used for treatment of chronic inﬂammatory diseases, cerebral edema, chronic pain syndrome, gastrointestinal diseases, tumors, as well as enhancing intelligence. Many studies have been carried out to discover therapeutic approaches for neurodegenerative diseases such as Alzheimer’s diseases, Parkinson’s disease, Huntington’s disease, multiple sclerosis and amyotrophic lateral sclerosis, stroke, and concomitant cognitive deficits. However, no curative treatment has been developed. This paper provides an overview of evidence about the potential of the Boswellia species and their main constituents, boswellic acids, as modulators of several mechanisms involved in the pathology of the neurodegenerative diseases. *In vitro*, animal, and clinical studies have confirmed that Boswellia species contain bioactive components that may enhance cognitive activity and protect against neurodegeneration. They exert the beneficial effects via targeting multiple pathological causes by antioxidative, anti-inflammatory, antiamyloidogenic, and anti-apoptotic properties. The Boswellia species, having neuroprotective potential, makes them a promising candidate to cure or prevent the neurodegenerative disorders.

## Introduction

**Figure 1.**
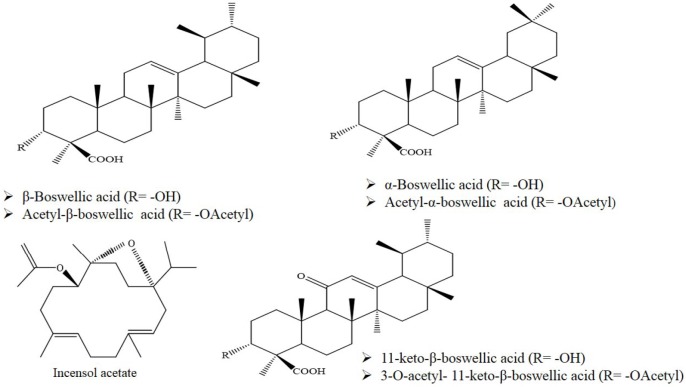
Chemical structure of main constituents of genus *Boswellia*

Neurodegenerative diseases such as Alzheimer’s disease (AD), Parkinson’s disease (PD), Huntington’s disease (HD), multiple sclerosis (MS), amyotrophic lateral sclerosis, and stroke are age-related disorders (1, 2). A number of common pathophysiological features have been proposed for these diseases, including elevated oxidative/nitrosative stress, mitochondrial dysfunction, protein misfolding/aggregation, synapse loss, and decreased neuronal survival (3, 4). Considering limitation of effective treatments for these diseases, there is an urgent need for new strategies using natural products that act through novel biological targets (5). The genus *Boswellia* belonging to the Burseraceae family comprises about 20 species. Species include *B. serrata*, *B. sacra*, *B. frereana*, *B. neglecta*, *B. microphylla*, *B. papyrifera*, *B. ogadensis*, *B. pirottae*, *B. rivae*, *B. madagascariensis*, *B. socotrana*, *B. popoviana*, *B. nana*, *B. ameero*, *B. bullata*, *B. dioscoridis*, *B. elongata*, and *B. ovalifoliolata*. *B. neglectae*, and *B. dalzielii *(6, 7). The genus is widespread in dry areas such as Arabia, northeastern coast of Africa, and India (8). The species have been useful in traditional medicine for treatment of inﬂammatory diseases, including asthma, arthritis, cerebral edema, chronic pain syndrome, gastrointestinal disease, tumors, and for enhancing memory and learning function (9-11). Frankincense, oleo-gum resins obtained from the genera *Boswellia*, is composed of essential oil (5-9%), mucopolysaccharides (20-23%), and resin (60%) (12, 13). The resinous part contains tetracyclic and pentacyclic triterpene acids. Boswellic acids (BAs) are considered the main biologically active components among the triterpene acids (Figure 1) (8, 14). Frankincense is responsible for anti-inﬂammatory and anti-cancer eﬀects of BAs (15, 16). The anti-inﬂammatory mechanisms are applied through inhibition of 5-lipoxygenase, cathepsin G, and microsomal prostaglandin-E synthase (mPGES)-1 (17). Other mechanisms include suppression of nuclear transcription factor κB (NF-κB) and pro-inﬂammatory cytokines such as tumor necrosis factor (TNFα), interleukin (IL)-1β, IL-2, and IL-6 (15, 17). Also, BAs lead to induction of apoptosis in cancer cells via activation of caspase-8 and inhibition of topoisomerases-I and II-alpha (16, 18). In this review, the therapeutic effects of Boswellia and their major constituents on various neurodegenerative disease models have been summarized (Figure 2). Herein, pharmacological effects of the genus Boswellia in neurodegenerative diseases were classified as follows:

1. Alzheimer’s disease

2. Parkinson’s disease

3. Cognitive dysfunction

4. Multiple sclerosis

5. Central nervous system trauma and brain ischemia

## Methods

To prepare this review, an online search was performed by using some databases, including PubMed, Google Scholar, Science Direct, and Scopus. This review mainly focuses on the therapeutic/or pharmaceutical effects of genus Boswellia and its main active constituents, AKBA, on neurodegenerative diseases (such as AD, PD, MS, and cerebral ischemia). The search terms included “Neuropharmacology”, “Learning”, “Memory”, “Neurocognitive”, “Neurodegenerative”, “Neurological disorders”, “Alzheimer’s disease”, “Parkinson’s disease”, “Multiple sclerosis”, “Cerebral ischemia”, “Boswellia”, and “AKBA (3-acetyl-11-keto-β-boswellic acid)”

**Alzheimer’s disease**

Alzheimer’s disease is the most common type of neurodegenerative dementia in older people (19). It is characterized by amyloid-beta (Aβ) accumulation in plaques and hyper-phosphorylation of tau protein forming neurofibrillary tangles (20). Aβ aggregation and neurofibrillary tangles induce neuron and synapse loss and gross degeneration in the temporal lobe, parietal lobe, as well as parts of the frontal cortex and cingulate gyrus (21). The pathological alterations cause progressive memory loss, cognitive impairment and the inability to perform daily activities (21, 22). Aβ toxicity, cholinergic dysfunction, oxidative damage, apoptosis, synaptic dysfunction, and senile plaque-induced inflammation have been postulated to be involved in pathogenesis AD (21, 23). The possible prophylactic and therapeutic effects of *B. serrata* using an animal model AD induced by AlCl_3_ (17 mg/kg for 4 weeks, orally) were assessed. In this study, rivastigmine (0.3 mg/kg/day), as standardized medicine, and *B. serrata *(45 and 90 mg/kg/day) were given for 2 weeks before AlCl_3_ administration to rats. The results revealed that activity of rats increased, while the duration taken by rats to reach food in the T-maze test decreased. According to biochemical analysis, treatment with *B. serrate* led to elevation of acetylcholine (ACh) levels while acetylcholine esterase (AChE) activity was suppressed in brain homogenates. The histopathology findings indicated that amyloid plaques reduced in the hippocampus (24). In a preclinical investigation, therapeutic potential of *B. serrata* against neurodegeneration using an AlCl_3_-induced rat model of AD was claimed. Following treatment of AD animals with *B. serrata* as resin methanolic extract (137.5 mg/kg, 3 months, orally), Aβ plaques in histopathological samples disappeared. Biochemical analysis showed brain and serum levels of AChE, C-reactive protein (CRP), nuclear factor kappa-light-chain-enhancer of activated B cells (NF-kB), monocyte chemoattractant protein-1 (MCP-1), and leukotriene B4 (LTB4) were suppressed while brain ACh and Bcl-2 were elevated. The data represented preventing efficacy of *B. serrata* against neuro-inflammatory and apoptosis insults (25). Also, co-administration of ginger (*Zingiber officinale*, 108 and 216 mg/kg) and *B. serrata* (45 and 90 mg/kg) in rats treated with AlCl_3_. The *B. serrata* and ginger improved histopathologic changes and also behavior stress tests, including activity cage, rotarod, T- maze, as well as restoring ACh and AChE levels in brain homogenate (26). Recent evidence revealed that insulin resistance and metabolic dysfunction play an important role in the pathology of sporadic Alzheimer’s disease (sAD) (27). Intracerebral-ventricular injection of streptozotocin (STZ, 2- deoxy-2-(3-(methyl-3-nitrosoureido)-D-glucopyranose) is applied to mimic sAD (28). STZ -induced insulin resistance causes several features characterizing AD including oxidative stress, neuroinflammation, and dysfunctions in adult neurogenesis that are followed by progressive deficits in learning and memory (29-31). A study explored whether aqueous extract of frankincense from *B. carteri* could have therapeutic effects on STZ- induced memory impairment. The evaluation of learning using passive avoidance task (PAT) indicated that chronic administration of aqueous extract of frankincense (50 mg/kg, 42 days) improved memory in rats receiving STZ (1.5 mg/kg/2 μl/side, i.c.v.) in a time-dependent manner (32). SuHeXiang Wan (SHXW) is a traditional Chinese medicine comprising *Liquidambar orientalis*, Saussurea lappa, Aquilaria agallocha, Santalum album, *B. carteri*, Eugenia caryophyllata, Cyperus rotundus, Styrax benzoin, and Dryobalanops aromatica that has been used orally for the treatment of seizures, infantile convulsions, and stroke (33). The potential beneﬁcial effects of SHXW essential oil were investigated on SH-SY5Y neuroblastoma cells and animal AD model induced by Aβ1-42 in mice. SHXW essential oil attenuated Aβ-induced cytotoxicity in SH-SY5Y cells through inhibition of apoptosis and ROS generation. Up-regulation of heme oxygenase-1 (HO-1), nuclear factor erythroid 2-related factor (Nrf2) expression, and increased Bcl-2/Bax protein ratio have been shown to be involved in the protective effects (34). SHXW essential oil ameliorated cognitive dysfunction in Aß1-42 treated mice, and it was associated with reduced p38, c-Jun N-terminal kinases, and tau phosphorylation (34). The findings suggested SHXW essential oil as a therapeutic agent for the prevention and treatment of AD and other tau protein pathology-related neurodegenerative diseases (34). Collectively, the experimental studies confirmed the inhibitory potential of Boswellia against formation of amyloid plaques and degeneration of cholinergic neurons induced by Aß. The medicinal herbs were found to induce anti-apoptotic activity through modulation of Bcl-2/Bax protein ratio. In addition, they counteracted oxidative damages through enhancement of HO-1/Nrf2 protein expression and restoring oxidative stress markers (Table 1).

**Parkinson’s disease**

Parkinson’s disease (PD), a common chronic, progressive neurodegenerative disorder of the elderly, is characterized by motor (including bradykinesia, tremor, and rigidity) and non-motor symptoms (35). The symptoms of PD result from the progressive degeneration of dopaminergic neurons in the substantia nigra pars compacta (SNpc) and dopamine (DA) deficiency in the striatum (36). Moreover, the presence of α-synuclein containing Lewy bodies in the surviving neurons is also proposed in the neuropathology of PD (37). Oxidative stress, mitochondrial dysfunction, excitotoxicity, calcium cytotoxicity, trophic factor deficiency, inflammatory processes, genetic factors, and apoptosis are now considered to be key mechanisms that contribute to neurodegeneration in PD (36). Some evidences has demonstrated the neuroprotective potential of *B. serrata* on dopaminergic neurons that can be applicable in PD. *Boswellia* resin extract (10 µg/ml) attenuated MPP+ (1-methyl-4-phenylpyridinium, 1000 µM), an active metabolite of 1-methyl-4-phenyl-1, 2, 3, 6-tetrahydropyryridine (MPTP)- induced toxicity in human dopaminergic SK-N-SH cell-line. The protective effects were associated with increased cell viability and reduced apoptotic features (38) (Table 1).

**Cognitive dysfunction**

Learning means the process of acquiring knowledge from the outside environment while memory is retention and retrieval of learned information at a later date (39-41). Short-term plasticity (STP) and long-term potentiation (LTP), two types of synaptic plasticity, are mechanisms for memory storage (42-45). Learning and memory impairment are considered the most significant features of dementia (46). A number of experimental studies were conducted to evaluate the effect of maternal administration frankincense (an oleo-gum resin derived from trees of genus Boswellia) on the cognitive capabilities. Hosseini Sharifabad *et al*. assessed learning and memory in two-month-old male Wistar rats whose mothers orally received aqueous extract of *B. serrata* (0.1 g/kg/day) during gestation (3 weeks) using active avoidance learning test. Frankincense enhanced power of learning at post-learning stage, short-term memory, and long-term memory (47). The results were relevant to the alteration in the neurites of CA3 hippocampal cells reported in another study. Analysis of morphology of dendritic architecture of CA3 hippocampal neurons indicated more dendritic segments and branching density in young rats whose mothers were treated with *B. serrata* (100 mg/kg/day) during gestation compared with untreated rats (48). Administration of aqueous extract of frankincense (50 and 100 mg/kg, 4 weeks) facilitated the learning and spatial memory formation in rats. The results were demonstrated as reduction in escape latency and traveled distance by the Morris water maze test method (49). An *in vivo* study was performed to assess the efficacy of frankincense for memory formation during development of the rat brain. For this purpose, aqueous extract of frankincense (50 and 100 mg/kg) was orally administered into female rats during gestation and lactation periods. Memory performance and hippocampal calcium/calmodulin kinase II (CaMKII) and CaMKIV mRNA levels in the offspring rats were evaluated to identify potential molecular change during gestation and lactation periods (50). CaMKII and CaMKIV are involved in many signaling cascades and are thought to be crucial mediators of learning and memory (51). CaMKII, as an important component of the postsynaptic density of glutamatergic synapses (52), plays a role in regulation of synaptic transmission and induction of long-term potentiation (LTP) (53). According to the results, up-regulation of CaMKII-α mRNA expression of the hippocampus was concomitant with improvement of spatial memory retrieval in oﬀspring rats (50). Evaluation of the spatial memory parameters by the Morris water maze (MWM) test revealed improvement of spatial learning and memory in rats treated with aqueous extract frankincense (50 and 100 mg/kg/day for 4 weeks). Frankincense up-regulated expression of brain-derived neurotrophic factor (BDNF) transcripts but not cAMP response element-binding (CREB). Therefore, the effects of the extract on memory formation may be attributed to another BDNF-related pathway other than BDNF–CREB–BDNF cycle (54). *B. papyrifera* total extracts (300 mg/kg, three times a day, orally) and boswellic acids fraction (100, 200, and 300 mg/kg) enhanced the retention phase of spatial memory of adult male rats in the MWM task. The results of the investigation proposed improving potential of Boswellia and boswellic acid fraction in memory function in normal subjects or neurodegenerative disorders (55). Impairment of cognitive function including memory, visuospatial organization, attention, and reaction time in overt hypothyroidism has been recognized for more than a century (56-58). Olibanum (resin of *B*. serrata) exhibited beneficial effects on memory deficit in methimazole-induced hypothyroidism model. Oral administration of olibanum (100 and 500 mg/kg, 180 days) improved memory and learning impairment in hypothyroid rats by the Morris water maze test (59). Animal models of amnesia induced by scopolamine are widely used to screen potential therapeutic value of compounds in treatment of dementia (60). Another study aimed to assess the effect of ethyl acetate and N-butanol fractions of *B. carterii* gum resin on intact memory and hyoscine-induced memory impairments using the MWM task. Ethyl acetate (0.1 mg/kg) and N-butanol (0.1 mg/kg) fractions remarkably enhanced intact memory. The ethyl acetate fraction was much more significant than other fractions in enhancing the memory (61). The combination of *Melissa oﬃcinalis* and *B. serrata* improved scopolamine-induced cognitive impairment. The MWM method revealed co-administration of M. oﬃcinalis and *B. serrata* (200 and 400 mg/Kg body weight) before scopolamine injection (0.1 mg/kg) led to improvement of memory function (62). Neuro-inflammation can cause cognitive deficits since it affects memory processing during consolidation and retrieval stages (63, 64). Considering anti-inflammatory activity of frankincense has been approved with an (15). Lipopolysaccharide (LPS) triggers the neuro-inflammatory process through activation of nuclear factor kappa B (NF-κB) pathway in microglia in the central nervous system (CNS) (65, 66). Administration of hydro-alcoholic extract of frankincense (50 mg/kg; orally) before LPS (1 mg/kg; IP) enhanced step-through latency (STL) in a passive avoidance task (PAT) via decreasing the TNF-α level in the hippocampus. Therefore, anti-inflammatory effects of frankincense may be involved in inhibition of memory loss (67). Clinical and pre-clinical studies have shown that prolonged frequent seizures cause cognitive, memory, and emotional impairments (68). These recurrent seizures affecting the hippocampus may lead to cell damage and death in the cornu ammonis (CA1) region (69). Function of CA1 neurons in the hippocampus plays a vital role in converting short-term memory to long-term memory (70). Pentylenetetrazol (PTZ)-induced kindled rats animal model was used for evaluation of epilepsy and its consequences on memory (71). The aqueous extracts of *B. serrata* (0.1, 0.5, and 1 g/kg, IP) improved passive-avoidance learning ability in kindled animals indicated by using shuttle box apparatus and step-through latency method. The findings were associated with increasing number of pyramidal neurons and dendritic spines in CA1 (71, 72). Therefore, consumption of *B. serrata* may be a therapeutic strategy for decreasing harmful effects of seizures on cognitive function (71). Age-related spatial learning deficits have been suggested to be due to changes that appear mostly in hippocampal connectivity and plasticity (73). The three main fields of the hippocampal region, CA1, CA3, and particularly dentate gyrus are vulnerable to aging (73). An experimental study conducted by *Hosseini-Sharifabad* *et al*. investigated the effects of chronic administration of *B. serrata* hydroalcoholic extract (BSE) on the learning performance and the morphology of hippocampal granule cells in aged rats (74). The rats (24 months old) received the aqueous extract of BSE (100 mg/kg/d, for 8 weeks, intragastrically), after this time, dendritic complexity in the dentate granule cells and spine density on the dendritic tree of the cells increased (74). These findings were observed along with improvement of spatial learning capability indicated as decrease in escape latency and swimming distance (74). Neuroprotective potential of Boswellia resin in age-related morphological changes and concomitant cognitive deficits may suggest it as a therapeutic agent in neurodegenerative diseases (74). In a randomized, parallel, double-blind, placebo-controlled clinical trial, administration of *B. serrata* and *Melisa oﬃcinalis* extracts (290 mg and 27 mg, for a month) improved memory in 70 older adults (75). Overall, this evidence provides preliminary support for the cognitive-enhancing efficacy of genus Boswellia. Potential beneficial actions may be attributed to BDNF up-regulation (Table 1). 

**Multiple sclerosis**

 Multiple sclerosis (MS) is a chronic autoimmune, inflammatory neurological disease of the CNS, which leads to the destruction of myelin, oligodendrocytes, and axons (76). Quinolinic acid (2, 3-pyridine dicarboxylic acid), a neuroactive metabolite of the kynurenine pathway, is an agonist of N-methyl-D-aspartate (NMDA). Inappropriate activation of the kynurenine pathway may increase quinolinic acid levels, which is often implicated in the pathogenesis of a number of neurological diseases such as MS (77, 78). *In vitro* study revealed that 24 hr pre-treatment of oligodendroglia (OLN-93) cells with ethanolic extract of* B. serrata* oleo-gum resin (10, 20, 40, and 80 µg/ml) prior to glutamate exposure reduced glutamate and quinolinic acid-induced oxidative injury (8 mM)(79). A mixture extract of *Portulaca olerace*, *Urtica dioica*, and *B. serrata* (200 and 400 mg/kg) has protective effects against ethidium bromide-induced MS model (80). The results revealed neurogenesis and memory improvement using the shuttle box test following treatment with the mixture (81). Cognitive deficits have been reported in up to 70% of MS patients (80). A clinical trial study was carried out in 80 patients with relapsing-remitting MS. In this randomized, double-blind, placebo-controlled study, effect of *B. papyrifera* on cognitive impairment in MS patients was investigated using brief international cognitive assessment for MS (BICAMS), symbol digit modality test (SDMT), and the California verbal learning test (CVLT). The patients received *B. papyrifera* (300 mg capsule, twice a day) and placebo with the same dose for 2 months. *B. papyrifera *remarkably improved visual-spatial memory while it was not effective in verbal memory and information processing speed, which may be due to the prescribed dose (82). Another double-blind clinical trial was carried out in 60 MS patients with cognitive deficits to assess the efficacy of *B. serrata* extract in treatment of cognitive dysfunction. The patients categorized randomly into two groups of treatment and placebo received 450 mg of *B. serrate* extract or placebo capsules, respectively, twice a day for two months. The extract remarkably enhanced auditory/verbal and visual/spatial memory using the brief visuospatial memory test (BVMT) and CVLT compared with the placebo group, which confirmed potential of *B. serrata* for MS patients suffering from cognitive impairments (83) (Table 1).

**Central nervous systems trauma and brain ischemia**

Stroke is the fourth cause of death and one of the main causes of disability worldwide (84). Ischemic stroke is the most common type of stroke, accounting for about 80 percent of all strokes, which results from transient or permanent cessation of cerebral blood flow (85, 86). Following brain ischemia, the level of glutamate increases, leading to over-activation of its receptors, including NMDA receptors and raised intracellular calcium (87). Brain ischemia also can trigger inflammatory responses and subsequently neuro-inflammation. Therefore, strategies targeting these pathways involve NMDA receptor antagonists, calcium channel blockers, and anti-inflammatory and antioxidant agents, which may be used as prophylactic or therapeutic for ischemia damage of brain tissue (87, 88). *B. serrata* and its constituent, AKBA (3-acetyl-11-keto-β-boswellic acid), exhibited potential neuroprotective and anti-oxidant activity (89). Neuroprotective potential of BSE and AKBA against ischemia-induced cytotoxicity was investigated. The survival of PC12 neural cells, pretreated with BSE (1.5-6 µg/ml) and AKBA (0.5–2.5 µg/ml) for 2 hr before exposure to oxygen/glucose/serum deprivation (OGSD) condition, increased. Moreover, BSE and AKBA counteracted oxidative stress indicated as restoring of intracellular reactive oxygen species content, lipid peroxidation, and oxidative DNA damage (90). Pre- and co-treatment with BSE and AKBA prevented glutamate-induced PC12 and Neuro-2a cell toxicity. The protective effect may be related to their inhibitory effects against oxidative damage and apoptotic cell death (91). An *in vitro* investigation aimed to explore anti-glycation and anti-oxidant potentials of *B. sacra* oleo-gum resin. Dichloromethane (CH_2_Cl_2_) fraction of the resin, 40% dichloromethane (CH_2_Cl_2_)/n-hexane sub-fraction, and frankincense oil exhibited l,l-Diphenyl-2-picrylhydrazyl (DPPH) free radical scavenging activity. In addition, moderate superoxide anion scavenging activity was exhibited by polar fraction, while the highest anti-glycation activity for polar fractions were reported (92). Another study was designed to investigate phytochemical screening, *in* *vitro* antioxidant activity of leaf extract of *B. serrate*. The methanolic extract of *B. serrata* contains alkaloids, terpenoids, saponins, and flavonoids (93). Methanolic extract exhibited significant DPPH free radical scavenging activity (IC_50_ = 54.06 µg/ml) and ferric reducing power (IC_50_ = 62.12 µg/ml), in a dose-dependent manner (93). Administration of the aqueous and ethanolic extracts of *B. serrat*a (125, 250, and 500 mg/kg, IP) and AKBA (50 mg/kg, IP) just after middle cerebral artery occlusion (MCAO), for 30 min and reperfusion for 24 hr improved neurological deficits and reduced brain infarction volume. The extracts diminished neuronal apoptotic cell death through up-regulation of Bcl-2 and down-regulation of Bax and caspase-3. The modulated cerebral redox status was also indicated as inhibition of lipid peroxidation while increasing glutathione content and superoxide dismutase activity in the cerebral cortex (94). The neuroprotective effects of Boswellia against brain stroke were further confirmed by the reduction of infarction volume and neurological impairments. The aqueous extract of frankincense was administrated (50, 100, and 150 mg/kg, orally for 30 days). Two hours after the last treatment with frankincense extract, the rats were subjected to MCAO for 60 min followed by reperfusion for 24 hr. The level of blood-brain barrier (BBB) permeability and stroke-induced brain edema decreased in rats treated with aqueous extract of frankincense at doses of 100 and 150 mg/kg (95). The results of a prospective, randomized, placebo-controlled, double-blind, pilot trial confirmed the efficacy of BS on cerebral edema following brain radiotherapy (96). In this trial, forty-four patients with primary or secondary malignant cerebral tumors randomly received BS (4200 mg/day) or placebo during radiotherapy. Administration of BS suppressed the edema volume which was evaluated by T2-weighted magnetic resonance imaging (MRI) (96). To investigate the effect of *B. serrata *on neuro-recovery following diffuse axonal injury (DAI), a double-blind, randomized, cross-over study was designed. The outcome of diffuse axonal injury was assessed using the disability rating scale, a surrogate clinical marker for the pace of neuro-recovery. 38 patients randomly received either placebo or BS capsules (containing 215 mg BS gum resin) for 6 weeks. The BS resin enhanced the cognitive outcome of patients with DAI (97). The protective effects of the condor against cerebral inflammation after induction of diffuse traumatic brain injury were investigated. *B. serrata* (250 and 500 mg/ kg) attenuated brain edema and disruption of blood-brain-barrier induced by traumatic brain injury. The results were accompanied by improvement of vestibulomotor dysfunction as well as modulation of IL-1β and I-10 in brain tissue. Anti-inflammatory properties were suggested to be involved in the neuroprotective effects (98). Boswellia exhibited therapeutic potential for brain ischemia and injuries, which is most likely related at least in part to its anti-inflammatory, anti-apoptotic, as well as anti-oxidative and free radical scavenging activities (Table 1). 

In this review, Tables 1 and 2 represent the brief description of pre-clinical and clinical studies on protective effects of genus Boswellia and AKBA in the neurodegenerative diseases, respectively.

**Table 1:** A summary of pre-clinical and clinical studies on protective effects of genus *Boswellia* in the neurodegenerative diseases

**Protective agent/dose/reference**	**Study design or experimental model**	**Main results**
*B. serrata *(45 and 90 mg/kg/day, 2 weeks) (24).	Animal model AD induced by AlCl_3_ in rat	Elevated ACh, suppressed AChE activity, improved histopathology changes, and reduced Aβ plaques in the hippocampus
*B. serrata* resin methanolic extract (137.5 mg/kg, 3 months) (25).	Animal model AD induced by AlCl_3_ in rat	Induced anti-neuro-inflammatory and anti-apoptotic properties indicated by suppression of serum level of AChE, CRP, NF-kB, MCP-1, LTB4, and elevation of brain ACh and Bcl-2. Aβ plaques disappeared
Co-administration of ginger (*Zingiber officinale*, 216 mg/kg) and *B. serrata* (45 and 90 mg/kg) (26).	Animal model AD induced by AlCl_3_ in rat	Improved histopathologic changes and behavior stress tests including activity cage, rotarod, and T- maze as well as restored ACh and AChE level in brain homogenate
Frankincense aqueous extract (50 mg/kg, 42 days) (32).	STZ (1.5 mg/kg/2 μl/side, i.c.v) - induced memory impairment	Evaluation of learning using passive avoidance task and improvement of memory
SHXW essential oil (1, 10, 100 µg/ml) (34).	SH-SY5Y neuroblastoma under Aβ1-42 (25 µM) toxicity	Attenuated Aβ-induced cytotoxicity through inhibition of apoptosis and ROS generation Up-regulated HO-1 and Nrf2 expression and Bcl-2/Bax protein ratio
	Mouse AD models induced by Aβ1-42	Ameliorated cognitive dysfunction in mice associated with reduced p38, c-Jun N-terminal kinases, and tau phosphorylation
*Boswellia* resin extract (10 µg/ml) (38).	An* in vitro* PD model induced by MPTP in human dopaminergic SK-N-SH cell-line	Attenuated MPTP-induced neurotoxicity including inhibition of apoptosis
1) *B. serrata *aqueous extract (0.1 g/kg/day) (47) 2) *B. serrata* (100 mg/kg/day) (48).	Assessment of cognitive dysfunction in young Wistar rats whose mothers received Boswellia during gestation (3 weeks)	Induced more dendritic segments and branching density in the neurites of CA3 hippocampal cells
Frankinsense aqueous extract (50 and 100 mg/kg, 4 weeks) (49).	Assessment of learning and spatial memory in rats using Morris water maze test method	Facilitated the learning and spatial memory formation as reduction in escape latency and traveled distance
Frankincense aqueous extract (50 and 100 mg/kg) during gestation and lactation periods (50).	Assessment of the frankincense efficacy on memory formation during development of the rat brain	Enhanced memory performance and up-regulated CaMKII and CaMKIV mRNA levels in the hippocampus offspring rats
Frankincense aqueous extract (50 and 100 mg/kg/day, 4 weeks) (54).	Evaluation of the spatial memory parameters by MWM test	Improved spatial learning and memory and up-regulated expression of BDNF but not CREB
*Boswellia papyrifera* total extracts (300 mg/kg, three times a day) and boswellic acids fraction (100, 200, and 300 mg/kg) (55).	Assessment of spatial memory using MWM task	Enhanced the retention phase of spatial memory proposing the improvement of memory function
Olibanum (100 and 500 mg/kg, 180 days) (59).	Assessment of memory function using methimazole-induced hypothyroidism animal model	Counteracted memory deficit in the Morris water maze test
Ethyl acetate (0.1 mg/kg) and N-butanol (0.1 mg/kg) fractions of *B. carterii* gum resin (61).	Memory impairments induced by hyoscine-induced	Ethyl acetate fraction was much more significant than other fraction in enhancing the memory ability indicated by the MWM task
Combined administration of *M. oﬃcinalis* and *B. serrata* (200 and 400 mg/kg) (62).	Spatial memory against cognitive impairment related to scopolamine	Improved memory performance indicated by MWM method
Frankincense hydro-alcoholic extract (50 mg/kg) (67).	Memory loss following LPS administration (1 mg/kg)	Enhanced step-through latency in a passive avoidance task accompanied by reduced TNF-α level in the hippocampus
Aqueous extracts of *B. serrata* (0.1, 0.5, and 1 g/kg, IP) (71, 72).	Pentylenetetrazol-induced kindled rats were used to study epilepsy and its consequences on memory using shuttle box apparatus and step-through latency method	Improved passive-avoidance learning ability associated with an increase in the number of pyramidal neurons and dendritic spines in CA1
*B. serrate* aqueous extract (100 mg/kg/d, for 8 weeks) (74).	Age-related morphological changes of hippocampal granule cells and concomitant cognitive deficits in escape latency and swimming distance	Enhanced dendritic complexity in the dentate granule cells and spine density associated with improvement of spatial learning capability
A tablet containing *B. serrata* and *Melisa oﬃcinalis* extract (290 and 27 mg) (75).	A randomized, parallel, double-blind, placebo-controlled clinical trial performed among 70 older adults	Improved memory function
Ethanolic extract of* B. serrata* oleo-gum resin (10, 20, 40, and 80 µg/ml) (79).	Oligodendroglia (OLN-93) cell injury induced by glutamate and quinolinic acid	Attenuated oxidative stress
The extract mixture of *Portulaca olerace*, *Urtica dioica*, and *B. serrata* (200 and 400 mg/kg) (81).	MS model induced by intra-hippocampal injection of ethidium bromide (stereotaxic surgery) in rats	Induced neurogenesis and memory improvement in the shuttle box test
Capsule containing* B. papyrifera* (300 mg, twice a day, 2 months) (82).	A randomized, double-blind, clinical trial in MS patients	Indicated therapeutic efficacy for cognitive dysfunction as improved visual-spatial memory
Capsule containing* B. serrate* extract (450 mg twice a day, two months) (83).	A double-blind clinical trial in MS patients with cognitive deficits	Improved cognitive deficits indicated by the improvement of auditory/verbal and visual/spatial memory in brief visuospatial memory test and California verbal learning test
*B. serrata* hydroalcoholic extract (1.5-6 µg/ml) and AKBA (0.5-2.5 µg/ml) (90).	Ischemia-induced cytotoxicity in PC12 cells following exposure to oxygen/glucose/serum deprivation condition	Increased cell survival and counteracted oxidative stress (ROS, lipid peroxidation, and oxidative DNA damage)
BSE (25, 50, 100 μg/ml) and AKBA (5 μm) (91).	Cell culture model of neurodegeneration induced by glutamate toxicity in PC12 and Neuro-2a cell	Inhibited oxidative damage and apoptotic cell death
*B. serrata* methanolic extract **(**50, 100, 250, 500, 1000, and 2000 µg/ml) (93).	*In vitro* assessment of antioxidant and anti-inflammatory activity	Exhibited DPPH free radical scavenging activity (IC_50_ = 54.06 µg/ml), ferric reducing power (IC_50_ = 62.12 µg/ml) stabilization towards human red blood cell membrane stabilization
Boswellia aqueous and ethanolic extracts (125, 250, and 500 mg/kg, IP) and AKBA (50 mg/kg, IP) (94).	An animal model of ischemia, MCAO	Improved neurological deficits and reduced brain infarction volume, neuronal apoptotic cell death accompanied by up-regulation of Bcl-2 and down-regulation of Bax and caspase-3. Reduced oxidative stress (counteracted lipid peroxidation and restored glutathione content and superoxide dismutase activity) in the cerebral cortex
Frankincense aqueous extract (100 and 150 mg/kg, 30 days) (95). 95	MCAO surgery was performed to induce ischemia-reperfusion status	The level of blood-brain barrier (BBB) permeability and stroke-induced brain edema and reduction of infarction volume and neurological impairments
BS (4200 mg/day) during radiotherapy (96).	A prospective, randomized, placebo-controlled, double-blind, pilot trial in cerebral edema following brain radiotherapy	Suppressed the edema volume evaluated by T2-weighted magnetic resonance imaging (MRI)
*B. serrata* capsules (containing 215 mg gum resin) for 6 weeks (97).	Assessment of effect on neuro-recovery following diffuse axonal injury in a double-blind, randomized, cross-over study	Enhanced the cognitive outcome of patients with diffuse axonal injury
*B. serrata* (250 and 500 mg/ kg) (98).	Cerebral inflammation after induction of diffuse traumatic brain injury	Attenuated brain edema and disruption of blood-brain-barrier accompanied by improvement of vestibulomotor dysfunction and modulation of IL-1β and IL-10 in the brain tissue

Ach: Acetylcholine; AChE: acetylcholine esterase; Aβ: amyloid Beta; Bcl-2: B-cell lymphoma 2; BDNF: Brain-derived neurotrophic factor; CREB: cAMP response element-binding protein; CRP: C-reactive protein; DPPH: 2,2-diphenyl-1-picrylhydrazyl; IL-10: Interleukin 10; LTB4: Leukotriene B4; MCAO: middle cerebral artery occlusion; MCP-1: Monocyte Chemoattractant Protein-1; MPTP: 1-methyl-4-phenyl-1, 2, 3, 6-tetrahydropyryridine; MWM: Morris water maze; MS: Multiple sclerosis; NF-kB: nuclear factor kappa-light-chain-enhancer of activated B cells; ROS: reactive oxygen species content; SHXW: SuHeXiang Wan; CaMKII: calcium/calmodulin kinase II

**Table 2:** A summary of in vitro and animal studies on neuroprotective potential of AKBA in the neurodegenerative diseases

Agent	Type of study	Protocol	Results	Ref.
AKBA	*In vivo* (MCAO) *In vitro* (OGD in primary cultured cortical neurons)	20 mg/kg AKBA were given immediately after the onset of reperfusion Incubation with AKBA for 24 hr	Treatment of AKBA : -reduced infarct volumes and apoptotic cells -increased neurologic scores by elevating the Nrf2 and HO-1 expression in brain -In primary cultured neurons: -increased the Nrf2 and HO-1 expression, -protection against OGD-induced oxidative stress	(99).
AKBA	*In vivo* (MCAO) *In vitro* (OGD in primary cultured astrocytes)	KBA (25 mg/kg) applied 1 hr after reperfusion Incubation with KBA for 24 hr	-reduced infarct volumes and apoptotic cells -increased neurologic scores -decreased MDA levels -restored the superoxide dismutase (SOD) activity -increased the protein Nrf2 and HO-1 expression in brain tissues -increased the Nrf2 and HO-1 expression -protection against OGD-induced oxidative stress	(100).
AKBA	*In vivo* (cognitive impairment in mice induced by LPS)	dual therapy with AKBA (at a dose of 5 mg/kg, IP for 4 days) and celecoxib (at a dose of 30 mg/kg, IP for 7 days)	-reversed the behavioral and molecular changes -anti-inflammatory effect -antiglutamatergic effect -anti-amyloidogenic agent	(101).
AKBA	*In vivo* (kainic acid-induced excitotoxicity and oxidative and nitrosative damage in mice)	the effects of COX inhibitors (indomethacin, nimesulide, and rofecoxib) and a 5-LOX inhibitor (AKBA) and the combination of these inhibitors in this model	-AKBA, indomethacin, and nimesulide did not produce any change in the behavioral parameters -rofecoxib increased the latency of clonic movement and decreased mortality rate -the effect of AKBA + rofecoxib was significantly more marked	(102).
Nano formulation of AKBA	*In vivo* (MCAO) *In vitro* (OGD)	AKBA-NPs (containing AKBA 10 mg/kg), intravenously. OGD + AKBA-NP (10 mg/ml) treatment	AKBA-NPs had better: -brain delivery efficacy -neuroprotection in OGD and MCAO models -modulation of antioxidant and anti-inflammatory pathways	(103).
AKBA	*In vivo* (a single IP dose of LPS (0.8 mg/kg) was injected to induce cognitive dysfunction)	LPS-treated mice were administered for 7 days with AKBA(5 mg/kg, IP) or DEX (1 mg/kg, IP)	-AKBA and DEX reversed the behavioral dysfunction AKBA: -decreased P-IκB-α, miRNA-155 expression level, and carbonyl protein content -restored normal cytokine level -increased SOCS-1 expression level -showed anti-apoptotic and anti-amyloidogenic effects	(104).
AKBA	*In vivo* (young and aged mice)	Chronic administration of AKBA (100 mg/kg, p.o.) and nimesulide (2.42 mg/kg, p.o.) for 15 days	-enhanced the cognitive performance -decreased oxidative damage -reversed the aging-induced motor dysfunction	(105).
AKBA	*In vivo* (MCAO)	AKBA (50 mg/kg) was administered IP after MCAO induction	Improved neurological deficit -reduced brain infarction -decreased neuronal cell loss and apoptosis -attenuated lipid peroxidation -increased glutathione content and superoxide dismutase activity	(94).
AKBA	*In vitro* (glutamate toxicity induced in PC12 and N2a cells)	Co- and pretreatment with AKBA (5 mM) was done on PC12 and N2a cells under glutamate toxicity (8 mM)	-↓ROS -↓lipid peroxidation -↓superoxide dismutase activity -↓oxidative DNA damage	(91).

MCAO: middle cerebral artery occlusion; OGD: oxygen-glucose deprivation; AKBA: acetyl-11-keto-β-boswellic acid; Nrf2: nuclear factor erythroid 2-related factor; MDA: malondialdehyde; LPS: lipopolysaccharide; COX: cyclooxygenase; DEX: dexamethasone; ROS: reactive oxygen species

## Conclusion

Considering lack of effective therapy for clinical applications, pharmacologically active natural products, having neuroprotective activities are being focused, which makes them potential candidates for neurodegenerative disorders. The genus Boswellia has been suggested to target various molecular pathways involved in pathogenesis of neurodegenerative diseases. The genus regulates neurotrophic factors (including BDNF), apoptotic proteins (pro-apoptotic caspase-3 and anti-apoptotic bcl-2), and redox status. They were shown to be therapeutically effective at controlling inflammatory and cholinergic systems. Therefore, evidence suggests the importance of the genus in the prevention and treatment of neurodegenerative diseases even though further studies and clinical trials on these promising medicinal plants and their constituents should be strongly encouraged in the future.
